# Effect of Interfacial Strength on Mechanical Behavior of Be/2024Al Composites by Pressure Infiltration

**DOI:** 10.3390/ma16020752

**Published:** 2023-01-12

**Authors:** Zeyang Kuang, Yixiao Xia, Guoqin Chen, Dongli Sun, Boyu Ju, Ping Wu, Wenshu Yang, Gaohui Wu

**Affiliations:** 1 School of Materials Science and Engineering, Harbin Institute of Technology, Harbin 150001, China; 2 Key Laboratory of Advanced Structure-Function Integrated Materials and Green Manufacturing Technology, School of Materials Science and Engineering, Harbin Institute of Technology, Harbin 150001, China; 3 Key Laboratory of Advanced Science and Technology on High Power Microwave, Xi’an 710024, China; 4 Northwest Institute of Nuclear Technology, Xi’an 710024, China

**Keywords:** Be/2024Al composites, pressure infiltration, interface strength, finite element analysis (FEA)

## Abstract

In this paper, two kinds of Be/2024Al composites were prepared by the pressure infiltration method using two different beryllium powders as reinforcements and 2024Al as a matrix. The effect of interfacial strength on the mechanical behavior of Be/2024Al composites was studied. Firstly, the microstructure and mechanical properties of the two composites were characterized, and then the finite element analysis (FEA) simulation was used to further illustrate the influence of interfacial strength on the mechanical properties of the two Be/2024Al composites. The mechanical tensile test results show that the tensile strength and elongation of the beryllium/2024Al composite prepared by the blocky impact grinding beryllium powder (blocky-Be/2024Al composite) are 405 MPa and 1.58%, respectively, which is superior to that of the beryllium/2024Al composite prepared by the spherical atomization beryllium powder (spherical-Be/2024Al composite), as its strength and elongation are 331 MPa and 0.38%, respectively. Meanwhile, the fracture of the former shows brittle fracture of beryllium particles and ductile fracture of aluminum, while the latter shows interface debonding. Further FEA simulation illustrates that the interfacial strength of the blocky-Be/2024Al composite is 600 MPa, which is higher than that of the spherical-Be/2024Al composite (330 MPa). Therefore, it can be concluded that the better mechanical properties of the blocky-Be/2024Al composite contribute to its stronger beryllium/aluminum interfacial strength, and the better interfacial strength might be due to the rough surface and microcrack morphology of blocky beryllium particles. These research results provide effective experimental and simulation support for the selection of beryllium powder and the design and preparation of high-performance beryllium/aluminum composites.

## 1. Introduction

Beryllium/aluminum composites, also known as Be–Al alloy, are actually aluminum matrix composites with beryllium phase as the reinforcement phase. Due to its excellent properties such as low density, high specific stiffness, specific strength, high thermal conductivity, low expansion, long range dimensional stability, and so on, beryllium/aluminum composites are very competitive in aerospace, national defense equipment, and other fields. At present, they are mainly used in optoelectronic systems, satellite structural components, thermal control systems, inertial navigation systems, and other fields [1,2,3].

**Figure 1:**
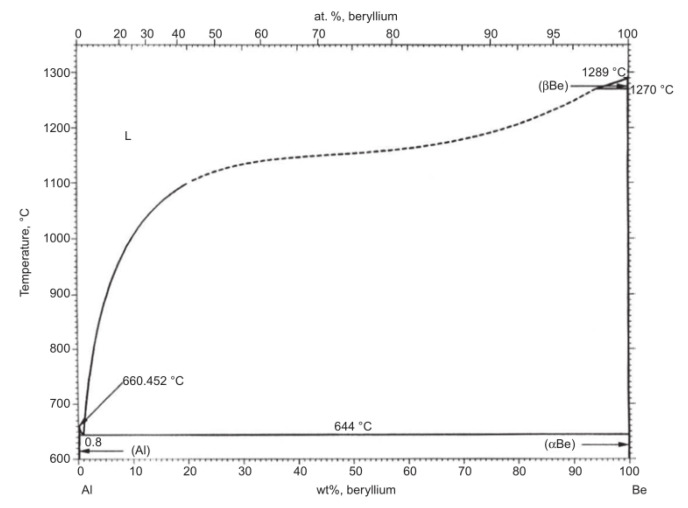
The Al–Be phase diagram [4]. Reprinted/adapted with permission from Ref. [4]. Copyright 1983, Springer Ltd.

The interfacial strength is one of the key factors affecting the properties of metal matrix composites. For beryllium/aluminum composites, it can be seen from the beryllium aluminum phase diagram [4] (Figure 1) that the solid solubility between the beryllium phase and the aluminum phase is very low, no intermediate phase or compound is formed, and the wettability between the beryllium phase and the aluminum phase is poor [5]. Therefore, it is difficult to form a strong interfacial bond between beryllium and aluminum without the influence of external factors, and it is hard to obtain beryllium/aluminum composites with high strength and plasticity. In general, interfacial bonding can be improved by means of surface treatment of reinforcements, addition of alloying elements (metal-based alloying), selection of preparation methods, and parameter control to increase the interfacial strength of composites [6,7,8,9]. According to different preparation methods, beryllium powder can be divided into disc-grinding powder, ball-grinding powder, impact-grinding powder, and gas atomization powder, etc. The characteristics of beryllium powder prepared by different preparation methods vary. The speed of ball milling and disc milling is slow, slips and fractures first occur on the {0002} basal plane of hexagonal beryllium crystal, which makes the particles mostly flake. The impact grinding process uses high-speed airflow to carry beryllium debris to impact on a beryllium target. The fracture of beryllium can also occur on other crystal planes other than the base plane, so that the particles are blocky, which can reduce the preferred orientation of beryllium products. Compared with the mechanical grinding method, the powders produced by the atomization method are typical spherical particles with high production efficiency, low cost, better fluidity, and higher filling density. [10,11]. The properties of beryllium or beryllium aluminum materials prepared by beryllium powder with dissimilar morphologies produced by various processes are different. Surface impurities can be removed by pickling beryllium powder and a relatively rough surface can be obtained at the same time to improve interface bonding strength [12]. However, surface treatment of beryllium powder is risky due to its toxicity. Adding alloying elements to the aluminum matrix is an effective way to obtain a uniform and refined microstructure, while helping to improve the interfacial strength between beryllium and aluminum, such as adding Mg, Si, Ag, Ge, Ni, Sc, Zr, etc. [13,14,15,16] At the same time, our previous work shows that in addition to powder metallurgy, precision casting, laser melting, and other preparation methods [17,18,19,20], the self-exhaust pressure infiltration technique has been successfully verified as an effective method to prepare high-strength Be/Al composites with good interfacial bonding [21]. Therefore, we selected two kinds of beryllium powders with different morphologies produced by different processes as reinforcements to prepare qualified beryllium/aluminum composites by the pressure infiltration method.

The specific interfacial strength of beryllium/aluminum composites is often difficult to obtain directly through experiments, while the FEA simulation can fit the interfacial strength value of the composites, and predict the toughness of the composite and analyze its fracture and damage mechanism on the basis of considering the microstructure of each phase of the composites [22,23,24,25]. Currently, 2D/3D modeling algorithms (3D reconstruction method [26], stochastic sequential adsorption algorithm [27], perturbation algorithm [28], Voronoi graph-based algorithm [29], etc.) and mechanical properties of particle-reinforced composites have been extensively studied in FEA simulation. Taking into account the complexity of establishing a 3D high-volume mechanical model and the computational efficiency, we used a 2D finite element model and an algorithm based on Voronoi diagrams to establish a multi-particle model close to the actual morphology and content of beryllium particles in order to estimate the interfacial strength of beryllium/aluminum composite to explain its fracture behavior.

Herein, blocky-Be/2024Al composite and spherical-Be/2024Al composite were prepared by pressure infiltration method using two kinds of beryllium powders with different morphologies: blocky impact grinding beryllium powder and spherical atomized beryllium powder. The microstructure and mechanical tensile properties of the two composites were characterized. The interfacial strength of Be/2024Al composites was simulated by FEA simulation for the first time, and the effect of beryllium powder morphology on the interfacial strength and mechanical behavior of Be/2024Al composites was studied.

## 2. Materials and Methods

### 2.1. Materials and Fabrication Process

**Figure 2:**
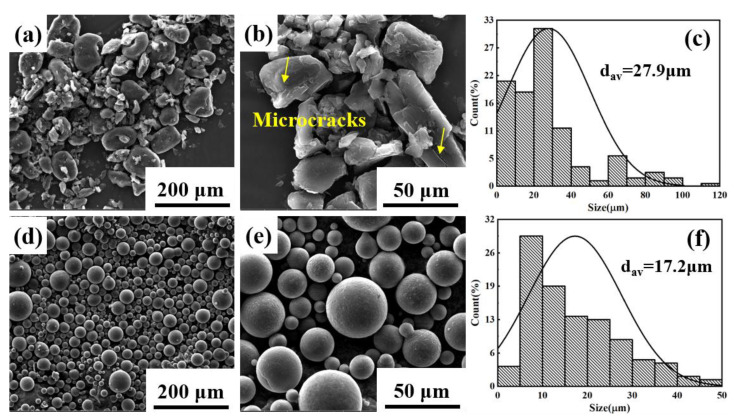
The morphology and size distribution of two kinds of Be powder. (**a**–**c**) Blocky impact grinding beryllium powder; (**d**–**f**) spherical atomized beryllium powder.

**Figure 3:**
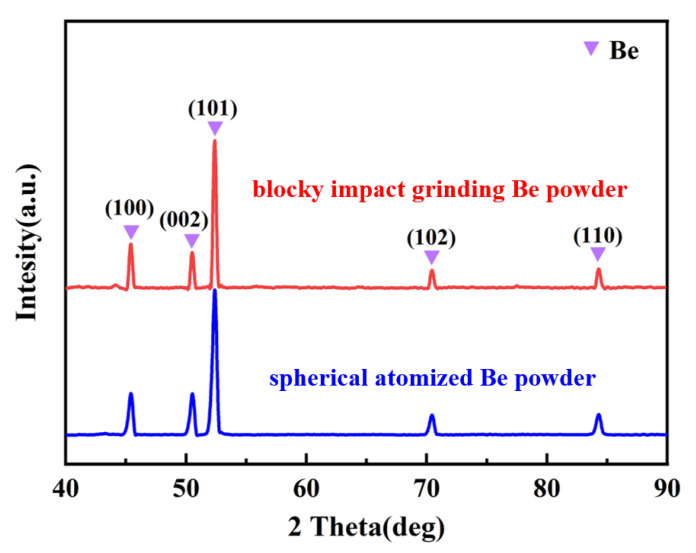
XRD pattern of two kinds of Be powder.

The blocky impact grinding beryllium powder and spherical atomized beryllium powder were used as the reinforcement, and the 2024 aluminum alloy was used as the matrix to prepare two kinds of Be/2024Al composites. The chemical composition of the raw materials is shown in Table 1. Figure 2 shows the morphologies and particle size distributions of the two kinds of beryllium powders. The beryllium powder prepared by impact grinding is blocky, there are triangular prism-like and round shape particles among the powders (Figure 2a), microcracks are observed on the surface of beryllium particles (marked with yellow arrows in Figure 2b), and the average particle size is about 27.9 μm (Figure 2c); the beryllium powder prepared by atomization has a regular spherical shape and a smooth surface (Figure 2d,e) with an average particle size of about 17.2 μm (Figure 2f). Figure 3 shows the XRD patterns of the two beryllium powders, neither of which has obvious impurity phase.

The fabrication process of the composites was the same as that of the Be/Al composites reported previously by self-exhaust pressure infiltration [21]. The beryllium powder was loaded into a steel mold in a vacuum glove box, and then pressed to a specified volume fraction to make a preform. The height of the preform was firstly calculated according to the preset volume fraction, and then the volume fraction of the preform was controlled by real-time observation of preform height and applied pressure. For blocky impact grinding beryllium powder, a preloading pressure of 5 MPa was applied to obtain a preform with a volume fraction of 53%. For the spherical atomized beryllium powder, due to the good fluidity of the spherical particles and the volume mismatch of the large and small particles, the preform was vibrated to a volume fraction of 63% without applying preloading pressure. The preform was placed in an argon-protected heating furnace at 10 °C/min to 600 °C for 1 h, while the 2024 aluminum alloy ingot was melted at 760 °C. Then the liquid aluminum was poured into the preform mold, and kept under the pressure of 10 MPa for 5 min to ensure the aluminum liquid fully infiltrated. Then the as-cast Be/2024Al composite was obtained by cooling and demolding. Before mechanical testing, all samples were subjected to T6 treatment (495 °C/1.5 h solution vacuum quencher, quenched in oil, 175 °C/10 h aging).

### 2.2. Characterization

The chemical composition of beryllium powder and 2024 aluminum alloy was obtained by ICP–AES (710-ES, Varian, Palo Alto, CA, USA) test. The morphology of beryllium powder, microstructure, and fracture characterization of the composites were performed on SEM (Quanta 200FEG, FEI, Hillsboro, MO, USA). Image J (V 1.8.0.112) software was used to calculate the particle size and distribution of Be particles based on SEM images. Before microstructure observation, the samples were successively ground with SiC sandpaper, coarsely polished with 0.25 μm diamond solution, and finely polished with saturated 0.02 μm SiO_2_ solution. The interface microstructure observation of Be/2024Al composites was performed on HRTEM (Talos F200X, FEI, Hillsboro, MO, USA) equipped with EDS. XRD test was used to characterize the phase composition of beryllium powder and composites. The XRD test was performed on Rigaku D/max-rB diffractometer (Rigaku, Tokyo, Japan) with the scanning range between 20° and 90° at the scanning speed of 2°/min. The tensile test was carried out on the Instron 5569 universal electrical testing machine. The test samples were dumbbell-shaped samples with gauge length of 15 mm and thickness of 2 mm, and the test speed was 0.5 mm/min. The Brinell hardness test was carried out on the Brinell hardness tester (LHBS-3000MD, YunCe, Kunshan, China). The sample size was 4 × 4 × 2 mm, the holding time was 30 s under the test force of 62.5 kgf. To improve the significance of the data, all mechanical tests were measured at least five samples.

### 2.3. Finite Element Model

**Figure 4:**
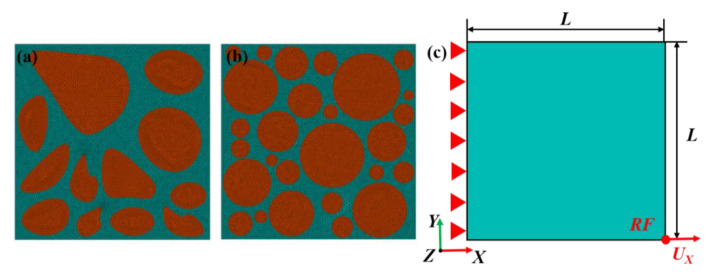
FEM model of Be/2024Al composites. (**a**) The mesh model of the blocky-Be/2024Al composite; (**b**) the mesh model of the spherical-Be/2024Al composite; (**c**) the loading direction of composites.

The 2D representative volume elements (100 μm × 100 μm) were used to represent the microstructure of the composites (Figure 4). A multi-particle model with a particle volume fraction of 53%, which was close to the actual microstructure of blocky-Be/2024Al composite, was established by an algorithm based on Voronoi diagrams. The model of spherical-Be/2024Al composite was drawn manually according to the actual microstructure, and the particle volume fraction was 63%. As for the finite element discretization, the matrix and beryllium particles were meshed by using the linear two-dimensional element CPE3 in ABAQUS. A 1 μm fine mesh discrete geometric model was used. Moreover, cohesive element (COH2D4) was inserted into the interface to explain the interface behavior between matrix and particles. These RVE models were generated in the XYZ Cartesian coordinate system. No periodic boundary conditions were applied. The fixed region X = 0 was fixed in 6 degrees of freedom, and the motion region X = L constrained by the reference point “RF” could only move along the loading X-direction on the U_X_ degree of freedom. A displacement loading of U = 0.05 L was applied on the “RF” (Figure 4c).

The Johnson–Cook constitutive model was used to describe the plastic deformation of the matrix [30]

(1) $$\bar{\sigma }=A+B\varepsilon^{n}$$

where $\bar{\sigma }\;$ represents the effective strain, *ε* represents the plastic strain, and *A*, *B*, and *n* represent the yield strength, strain hardening parameter, and hardening exponent of the matrix, respectively. Johnson–Cook damage model was also used to describe the fracture behavior of the matrix [31]

(2) $$D=\int \frac{1}{\varepsilon_{f}}d\dot{\varepsilon }$$

when the damage variable *D* reaches 1, the fracture occurs; $d\dot{\varepsilon }$ represents the equivalent plastic strain increment; $\varepsilon_{f}$ represents the equivalent strain at fracture, its expression is as follows:

(3) $$\varepsilon_{f}=D_{1}+D_{2}\exp(D_{3}\sigma^{*})$$

where *D_i_*(*i* = 1, 2, 3) are parameters determined by material properties, $\sigma^{*}$ represents the stress triaxiality. In order to define the deformation and fracture behavior of the matrix, a host of tensile and compressive tests were conducted on the 2024Al ingot obtained by pressure infiltration to obtain the parameters required for the J–C constitutive model, as listed in Table 2.

An element-based cohesion method was utilized to forecast the traction–separation behavior of the Be–Al interface. Quadratic stress criterion was used to determine the damage initiation the cohesive zone model (CZM) [32]:

(4) $${\left\{\frac{\left\langle t_{n}\right\rangle }{t_{n}^{0}}\right\}}^{2}+{\left\{\frac{t_{s}}{t_{s}^{0}}\right\}}^{2}+{\left\{\frac{t_{t}}{t_{t}^{0}}\right\}}^{2}=1$$

where $t_{n}$ represents the nominal traction stress in the normal direction, while $t_{s}$ and $t_{t}$ are the nominal traction stresses in the two shear (*n* and *t*) directions, respectively. The superscript 0 indicates the contact stress at the beginning of the damage. A variable $D^{\prime }$ was utilized to describe the damage evolution process [33], which was defined as:

(5) $$D^{\prime }=\frac{\delta_{m}^{f}\left(\delta_{m}^{max}-\delta_{m}^{0}\right)}{\delta_{m}^{max}\left(\delta_{m}^{f}-\delta_{m}^{0}\right)}$$

(6) $$\delta_{m}=\sqrt{\delta_{n}{}^{2}+\delta_{s}^{2}+\delta_{t}^{2}}$$

where

- $\delta_{m}$ is the effective displacement of damage evolution;
- $\delta_{n}$, $\delta_{s}$, and $\delta_{t}$ are nominal displacements in normal and two shear directions;
- $\delta_{m}^{0}$ and $\delta_{m}^{f}$ are the effective displacements at the damage initiation and the fracture completion, respectively;
- $\delta_{m}^{max}$ is the maximum effective displacement obtained during loading.

From the perspective of energy evolution, fracture energy *G_c_*, interface strength $T_{eff}^{0}$, and effective displacement at complete failure $\delta_{m}^{f}$ satisfied the following relations:

(7) $$G_{c}=\frac{1}{2}\delta_{m}^{f}T_{eff}^{0}$$

In addition to simulating interfacial debonding, the CZM model was also widely used in crack propagation and fracture of polycrystalline materials [34,35,36]. We used the CZM model to describe the fracture behavior of beryllium particles. The relevant performance parameters are listed in Table 3.

## 3. Results and Discussion

### 3.1. Microstructure and Phase Composition

**Figure 5:**
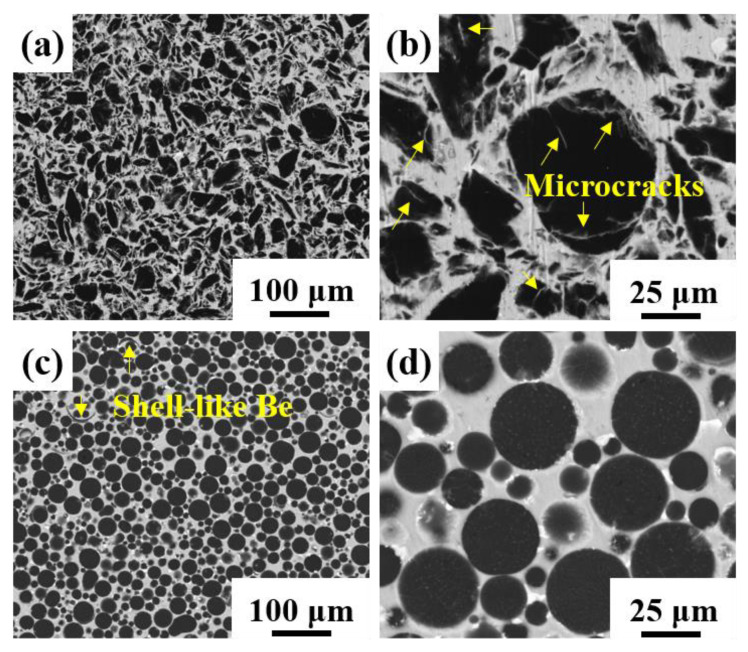
SEM photographs of two kinds of Be/2024Al composites fabricated with different beryllium powder: (**a**,**b**) blocky-Be/2024Al composite; (**c**,**d**) spherical-Be/2024Al composite.

**Figure 6:**
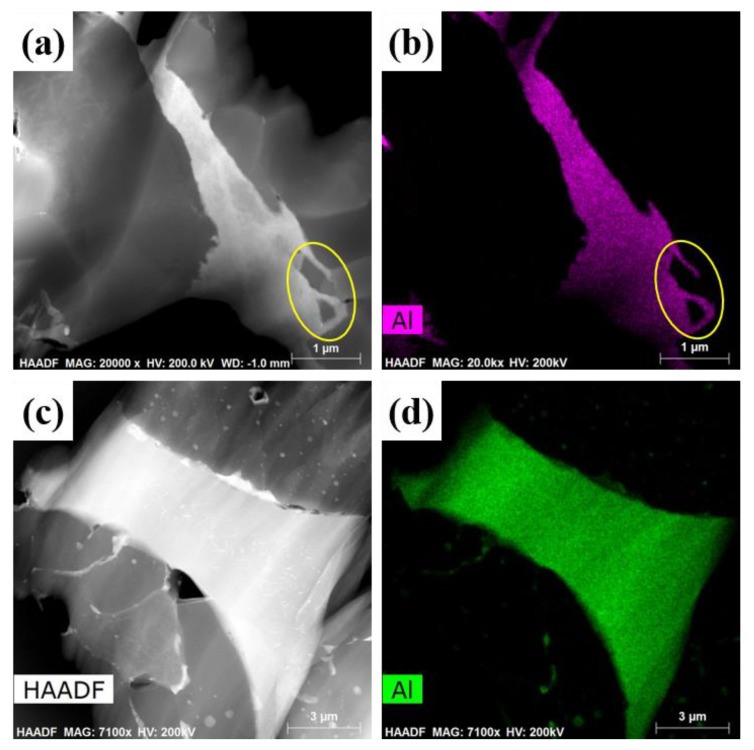
TEM photographs of two kinds of Be/2024Al composites fabricated with different beryllium powders. (**a**) Typical HAADF image of the blocky-Be/2024Al composite; (**b**) the corresponding Al element distribution of (**a**); (**c**) typical HAADF image of the spherical-Be/2024Al composite; (**d**) the corresponding Al element distribution of (**c**).

The microstructures of blocky-Be/2024Al composite and spherical-Be/2024Al composite are shown in Figure 5. The black beryllium particles are uniformly distributed in the off-white aluminum matrix, and no large area agglomeration of small particles, no holes, or other obvious defects are observed. As microcracks exist on the surface of blocky impact grinding beryllium particles (marked by yellow arrows in Figure 2b), off-white microcracks filled with aluminum can also be seen in the SEM image of blocky-Be/2024Al composite (labeled by yellow arrows in Figure 5b). However, in the spherical-Be/2024Al composite, most of the beryllium particles are regularly spherical with no apparent surface microcracks, except for one or two shell-like beryllium reinforcements due to the presence of a few hollow beryllium spheres (labeled by the yellow arrow in Figure 5c). The distribution of Al in the two Be/2024Al composites is shown in Figure 6. For the blocky-Be/2024Al composite, the liquid aluminum penetrates into the nanoscale microcracks of the blocky beryllium powder (marked by yellow circles in Figure 6a,b). For the spherical-Be/2024Al composite, a small amount of aluminum segregation exists inside and on the surface of beryllium particles. These segregations are not caused by the infiltration of aluminum during the preparation of the composite, but the aluminum impurities in the beryllium raw material itself, which can be seen from the high Al content of the spherical powder (1.1 wt%) in the elemental analysis results (Table 1).

**Figure 7:**
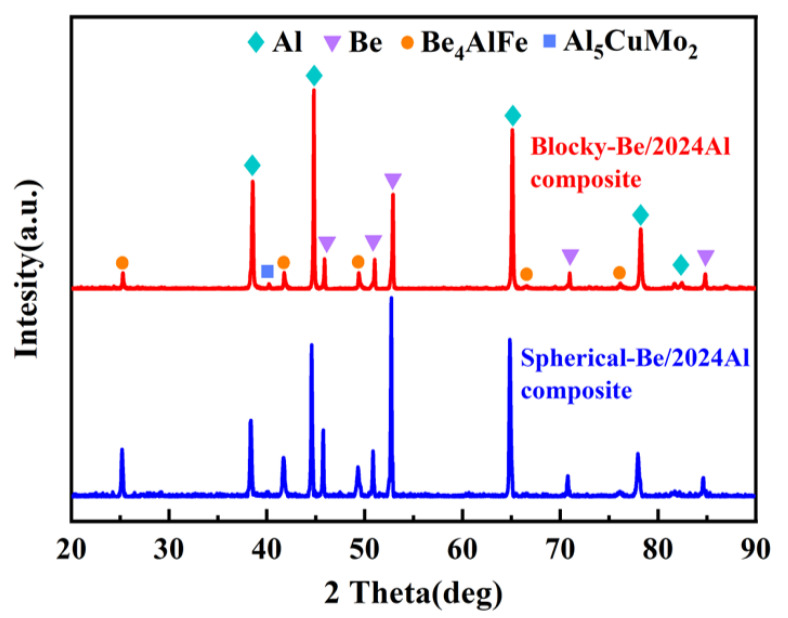
XRD pattern of two kinds of Be/2024Al composites.

In addition, in the SEM images of the microstructures of the two composites at high magnification (Figure 5b,d), a white impurity phase exists near the beryllium particles. According to the XRD phase composition analysis (Figure 7), it can be determined that the impurity phase in the two composites is the Be_4_AlFe phase, and the content of impurity phase in the spherical-Be/2024Al composites is higher. The formation of the Be4AlFe phase may be related to the Fe content of beryllium powder and Al matrix (Table 1). The Be_4_AlFe phase is a common impurity phase in beryllium. Fe can be solid dissolved in Be, and the binary beryllium iron phase can combine with free Al to form Be_4_AlFe [10]. Due to the relatively low content of the impurity phase in the raw beryllium powder, it cannot be detected by XRD. However, due to the introduction of Fe from the Al matrix, and the higher content of Fe in the spherical beryllium powder compared to that in the blocky beryllium powder, the Be_4_AlFe phase in the spherical-Be/2024Al composite is more than that in the blocky-Be/2024Al composite. The impurity phase that mainly exists at the interface of beryllium phase and aluminum phase may also affect the mechanical properties of the composites. Moreover, a slight Al_5_CuMo_2_ phase diffraction peak is observed in the XRD pattern. The Al_5_CuMo_2_ phase is the precipitated phase in the matrix. Since Mo does not exist in the matrix alloy and beryllium reinforcement, the Mo element may be the impure element introduced in the preparation process. Due to the small content, only the strongest diffraction peak of the Al_5_CuMo_2_ phase is detected in the XRD pattern.

### 3.2. Mechanical Properties and Fracture Analysis

**Figure 8:**
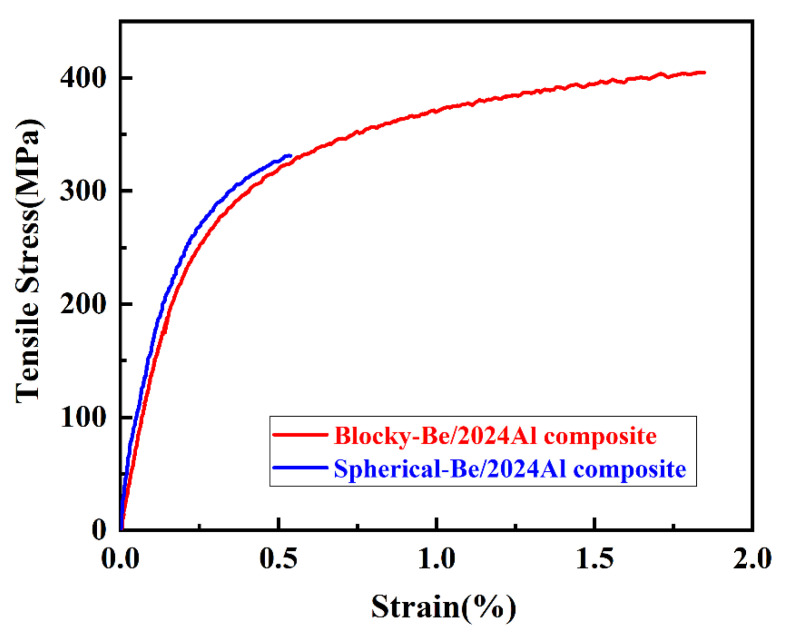
Representative tensile curves of two kinds of Be/2024Al composites.

Figure 8 shows the typical tensile curves of blocky-Be/2024Al composites and spherical-Be/2024Al composites. Table 4 lists the specific values of mechanical properties of these two composites. The hardness and yield strength of blocky-Be/2024Al composites are slightly lower than those of spherical-Be/2024Al composites. This is because the beryllium content of the blocky-Be/2024Al composites is slightly lower than that of the spherical-Be/2024Al composites. However, its tensile strength and elongation are 405 MPa and 1.58%, respectively, which are higher than 331 MPa and 0.38%, respectively, of the spherical-Be/2024Al composites.

**Figure 9:**
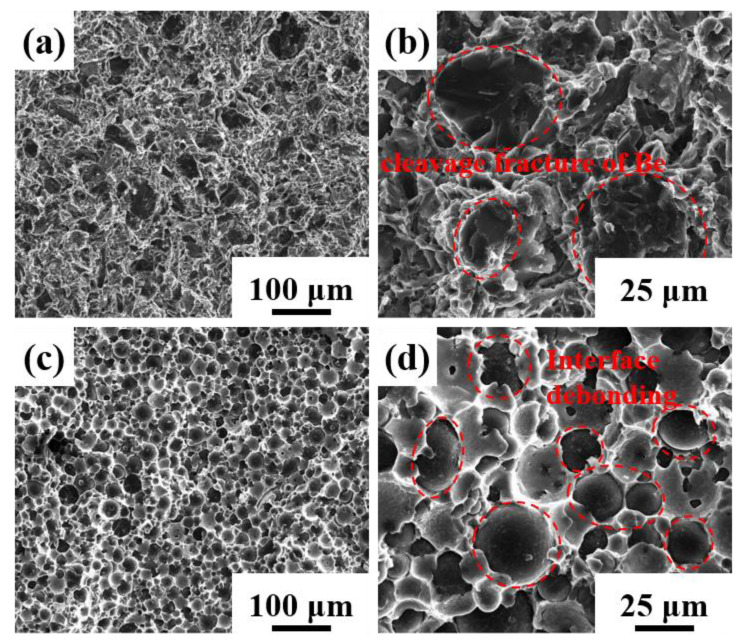
Fracture surfaces of different kinds of Be/2024Al composites. (**a**,**b**) Blocky-Be/2024Al composite; (**c**,**d**) spherical-Be/2024Al composite.

Figure 9 shows the typical fractures of the two composites. The blocky-Be/2024Al composites exhibit a mixed fracture mode of cleavage fracture of beryllium and ductile fracture of aluminum, and large particles of beryllium are more prone to cleavage fracture. The spherical-Be/2024Al composites show the fracture mode of interfacial debonding. It can be observed from the fracture that a large number of spherical beryllium particles are pulled out, exposing the smooth surface of beryllium particles (Figure 9d). Severe interfacial debonding is the principal cause for the lower strength and poor ductility of the spherical-Be/2024Al composites, and interfacial debonding is due to the low interfacial bonding strength. Therefore, the stress and strain distribution during the tensile process and the interfacial bonding strength of the two composites are further analyzed by FEA simulation.

### 3.3. Numerical Results

**Figure 10:**
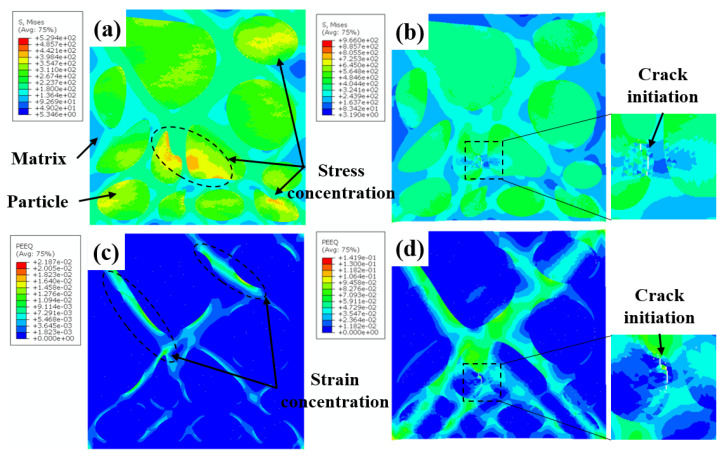
Distribution of the Mises stress (**a**,**b**) and the equivalent plastic strain (**c**,**d**) in the blocky-Be/2024Al composite under the nominal strain of (**a**,**c**) ε_n_ = 0.0027; (**b**,**d**) ε_n_ = 0.018.

Figure 10 shows the Mises stress and equivalent plastic strain distribution of the blocky-Be/2024Al composite at different strain stages. As shown in Figure 10a,c, in the initial stage of tension, the distribution of stress and strain is not uniform, and the high stress area is mainly concentrated on beryllium particles, especially cluster particles and particles with sharp corners (marked by the black dotted circle in Figure 10a). The strain is mainly concentrated in the matrix and presents an angle of about 45° from the loading direction, and the strain is also prominent at the particles with sharp angles. Subsequently, with the accumulation of deformation, beryllium particles begin to fail during the tensile process, forming microcracks (Figure 10d), and the stress on both sides of the crack is significantly released (Figure 10b).

**Figure 11:**
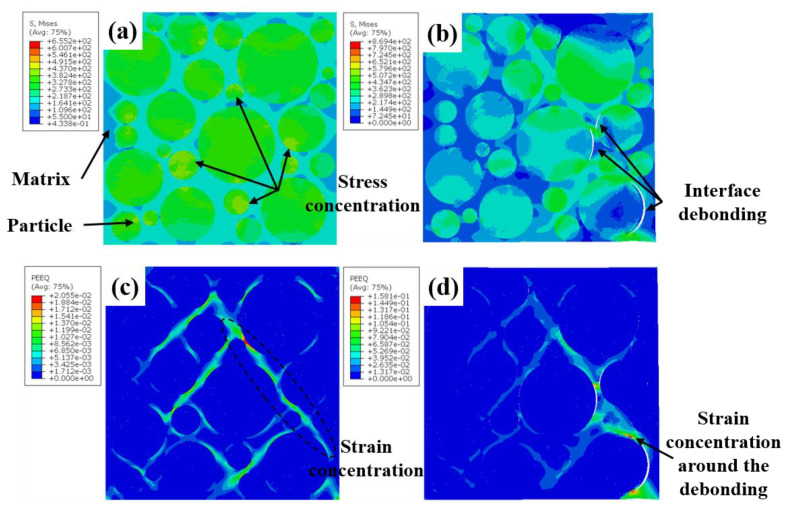
Distribution of the Mises stress (**a**,**b**) and the equivalent plastic strain (**c**,**d**) in the spherical-Be/2024Al composite under the nominal strain of (**a**,**c**) ε_n_ = 0.0027; (**b**,**d**) ε_n_ = 0.0078.

The Mises stress and equivalent plastic strain distribution of the spherical-Be/2024Al composites at different strain stages are shown in Figure 11. Similar to the blocky-Be/2024Al composites, in the initial stage, stress concentration of the composite is mainly in the beryllium particles, and the small particles bear higher stress (Figure 11a), while the strain is mainly concentrated in the aluminum matrix, the stress concentration zone and the loading direction is about 45°, and there is a high degree of strain concentration in the aluminum matrix between the adjacent beryllium spheres (Figure 11c). With the increase in deformation, debonding occurs at the beryllium–aluminum interface, resulting in microcracks, and the strain is highly concentrated along the debonding interface, which eventually leads to a complete debonding of the interface.

**Figure 12:**
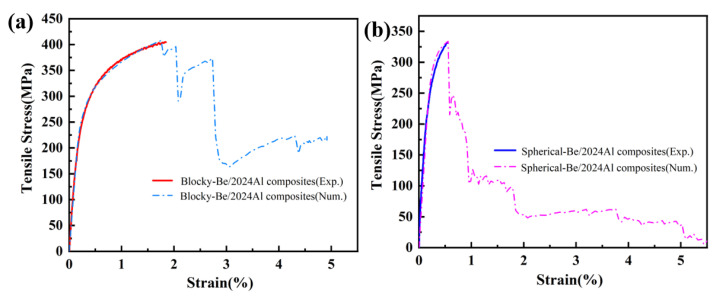
Comparison of the stress–strain curves between numerical and experimental results. (**a**) Blocky-Be/2024Al composite; (**b**) spherical-Be/2024Al composite.

In this simulation, we determined the interface strength by fitting the experimental nominal stress–strain curve. Figure 12 compares the nominal stress–strain curves of the two composites obtained from the tensile simulation with the experimental results. The results show that the simulated stress–strain curve of the blocky-Be/2024Al composite agrees well with the experimental results when the interfacial strength is 600 MPa (Figure 12a). When the interfacial strength is 330 MPa, the simulated stress–strain curve of spherical-Be/2024Al composite fits well with the experimental results (Figure 12b). Therefore, we can conclude that the interface strength of the blocky-Be/2024Al composite is 600 MPa and that of the spherical-Be/2024Al composite is 330 MPa.

**Figure 13:**
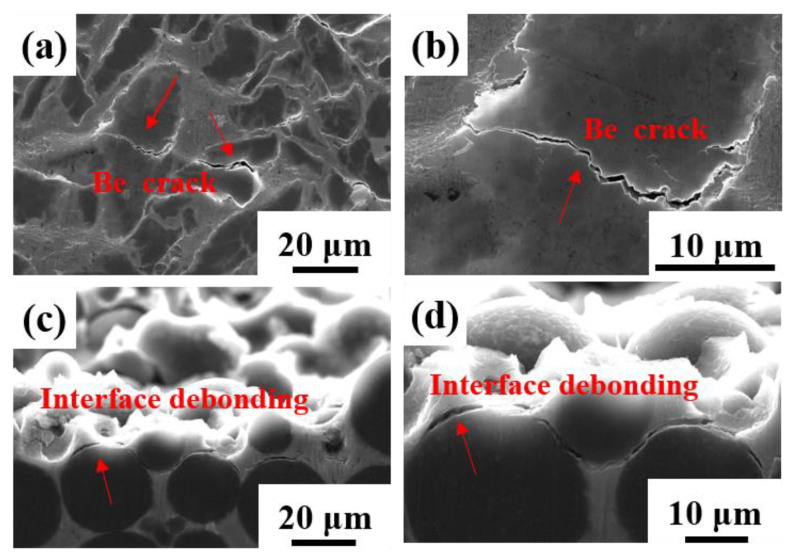
SEM photographs of the longitudinal section microstructure of the tensile fracture. (**a**,**b**) Blocky-Be/2024Al composite; (**c**,**d**) spherical-Be/2024Al composite.

Meanwhile, the cross-section of the tensile fracture specimens of the composites is observed, and the typical microstructures are shown in Figure 13. The microcracks in the blocky-Be/2024Al composite mainly appear inside the beryllium particles (Figure 13a,b); the microcracks in the spherical-Be/2024Al composite are mostly present at the beryllium–aluminum interface, and obvious debonding can be observed, while the beryllium particles are hardly broken (Figure 13c,d). These observed results are consistent with the simulation results in Figure 10 and Figure 11.

It can be seen that the difference in interfacial strength is the main reason for the variance in tensile strength and elongation of the two composites. The interfacial strength of the blocky-Be/2024Al composite is 600 MPa, which is much higher than that of the spherical-Be/2024Al composite (330 MPa). Although there are no reports on the specific experimental values or numerical simulation values of the interfacial bonding strength of beryllium–aluminum composites to judge the level of our interfacial strength values, we can obtain a glimpse from the reported interfacial strength values of SiC/Al composites calculated by the finite element CZM model. The interfacial strength values of SiC/Al composites given in different studies in the literature are slightly different. Su et al. reported that the interfacial strength of the extruded 13 μm SiC/7A04Al was 326 MPa [23], Zhang et al. found that the interfacial strength of 7 vol.% SiC/7A04Al was 400 MPa [24], Wu et al. reported that the interfacial strength of the as-cast 20 vol.% A359/SiC was 372 MPa [25], while the interfacial strength of the extruded 17 vol.% 7 μm SiC/2009Al composite was 600 MPa [22]. It can be seen that the reported interfacial strength of the SiC/Al composites is between 300–600 MPa, and is significantly related to the content, size, morphology, distribution of the reinforcement, and the mechanical properties of the matrix. In this study, the calculated interfacial strength of Be/2024Al composites is also within the reasonable range of the reported interfacial strength of SiC reinforced aluminum matrix composites, and the interfacial strength of the blocky-Be/2024Al composites is close to the highest reported interfacial strength of SiC reinforced aluminum matrix composites, which indicates that the blocky-Be/2024Al composites have high interfacial strength. What needs to be emphasized is that the difference in interfacial strength is related to the morphology of the reinforcement. Roughness influences the interface properties. Studies show that for concrete, the waviness of the concrete aggregate not merely increases the actual bonding area of the interface, it also enhances the mechanical interlocking between the matrix and the aggregate [37]. Similarly, the blocky beryllium powder has rough surface and microcracks, and the infiltration of aluminum in the microcracks of beryllium particles during pressure infiltration process can bring larger area mechanical bonding, which results in a stronger interfacial strength. The surface of the spherical beryllium particle is smooth, and liquid aluminum cannot penetrate into the beryllium sphere, meaning that the interface bonding between beryllium and aluminum is relatively weak. Therefore, the morphology of beryllium powder is one of the main factors affecting the interfacial strength of pressure infiltration Be/2024Al composites.

It cannot be ignored that the presence of the impurity phase Be_4_AlFe also affects the mechanical properties of Be/2024Al composites. The brittle interface impurity phase Be_4_AlFe easily becomes the stress concentration point, which leads to the premature fracture of Be/2024Al composites during the tensile process and reduces the ductility of the composites. The higher Be_4_AlFe content in the spherical-Be/2024 Al composite also makes it more prone to brittle fracture.

## 4. Conclusions

In this paper, blocky-Be/2024Al composites and spherical-Be/2024Al composites were fabricated by the self-exhaust pressure infiltration method. Their microstructures and mechanical properties were characterized. The fracture mode and interfacial strength of the two composites were fitted by FEA simulation. The main results are as follows:

1. The microstructures of the blocky-Be/2024Al composites and spherical-Be/2024Al composites prepared by self-exhaust pressure infiltration are uniform and dense. The tensile strength of the blocky-Be/2024Al composite is 405 MPa and the elongation is 1.58%. The tensile strength and elongation of the spherical-Be/2024Al composite are 331 MPa and 0.38%, respectively;
2. The tensile fracture modes of the blocky-Be/2024Al composites and spherical-Be/2024Al composites are different. The former shows brittle fracture of beryllium particles and ductile fracture of aluminum, while the latter shows interface debonding;
3. The results of FEA simulation show that the interfacial strength of the blocky-Be/2024Al composite is 600 Mpa and that of the spherical-Be/2024Al composite is 330 MPa. The fracture microstructure of the composites is consistent with the simulation results. The difference in strength and fracture mode of the two composites is mainly caused by the difference in interfacial strength, and the morphology of beryllium powder is one of the main reasons for the difference in interface strength;
4. The existence of the impurity phase Be4AlFe also affects the mechanical properties of the Be/2024Al composites. The brittle impurity phase Be_4_AlFe is likely to become a stress concentration point, resulting in premature fracture of the composites during the tensile process and reducing the ductility of the composites.

## Figures and Tables

**Table 1:** Composition of raw materials (wt.%).

Materials	Cu	Mg	Mn	Fe	Si	Zn	Cr	Ni	Ti	Al	Be
Blocky Be	0.005	<0.003	0.023	0.199	0.015	<0.003	0.032	0.007	--	0.023	Bal.
Spherical Be	0.003	0.034	0.044	0.292	0.044	--	--	0.038	--	1.100	Bal.
2024Al	4.89	1.57	0.64	0.15	0.20	0.19	0.01	--	0.04	Bal.	--

**Table 2:** Johnson–Cook parameters of 2024Al.

A (MPa)	B (MPa)	n	D_1_	D_2_	D_3_
185	2631	0.78	0.13	0.13	−1.5

**Table 3:** Material properties used in the REV model.

Properties	Be	2024Al
Density (g/cm^3^), ρ	1.85	2.77
Young’s modulus (GPa), E	300	70
Poisson’s ratio, ν	0.03	0.33
Tensile strength (MPa), σ_UTS_	505	289
Yield stress (MPa), σ_YS_	419	185

**Table 4:** Mechanical properties of two kinds of Be/2024Al composites.

Samples	Brinell Hardness (HB)	Tensile Strength (MPa)	Yield Strength (MPa)	Elongation (%)
Spherical-Be/2024Al	157.1 ± 2.15	331 ± 11.2	299 ± 8.5	0.38 ± 0.11
Block-Be/2024Al	148.2 ± 3.43	405 ± 9.3	297 ± 7.9	1.58 ± 0.22

## Data Availability

The data presented in this study are available on request from the corresponding author.
